# Treatment decision-making factors among patients with cervical myelopathy: a discrete-choice experiment

**DOI:** 10.1186/s41687-024-00810-z

**Published:** 2024-11-11

**Authors:** Mohamed Sarraj, Meerab Majeed, Mohammad Zarrabian, Jason Busse, Mohit Bhandari, Daipayan Guha, Markian Pahuta

**Affiliations:** 1 Division of Orthopedic Surgery, McMaster University, Hamilton General Hospital, Hamilton, ON Canada; 2 University of Toronto, Toronto, ON Canada; 3 Departments of Anesthesia and Health, Evidence and Impact, McMaster University, Hamilton, ON Canada; 4 Division of Neurosurgery, Hamilton General Hospital, McMaster University, Hamilton, ON Canada

**Keywords:** Cervical myelopathy, Shared decision making, Discrete choice experiment, mJOA

## Abstract

**Background:**

Degenerative Cervical Myelopathy is a debilitating condition and current recommendations encourage shared decision-making between surgeons and patients. However, there is limited data on patients’ values and preferences for surgical decision making. This study aimed to quantify and compare the relative importance of neurologic function, risk of future surgery, and complications to patients with cervical stenosis.

**Methods:**

Patients with cervical stenosis presenting for surgical evaluation, or post-operative cervical decompression patients, were recruited to participate. Demographic information including modified Japanese Orthopedic Association (mJOA) score, type of surgery, and complications were recorded and anonymized to study ID. Patients then completed an online discrete-choice experiment survey. In a series of 10 questions, respondents chose between two hypothetical health states defined in terms of five attributes, or “decision factors”: (i) upper extremity neurologic function, (ii) lower extremity neurologic function, (iii) risk of cervical spine surgery, (iv) dysphagia, and (v) C5 palsy. Participants were asked to choose which ‘life’ they preferred, and a regression model was used to quantify the importance of each decision factor.

**Results:**

We report three key findings that can aid clinicians in shared decision-making conversations: (i) all patients regard lower extremity neurologic function as the most important decision factor, (ii) dysphagia, a complication, and upper extremity neurologic function are equally important, and (iii) patients who have undergone surgery weigh neurologic function as less important, and complications as more important than patients who have not undergone surgery.

**Conclusions:**

Patient preferences for management of degenerative cervical myelopathy are influenced by several considerations including the experience of surgery itself. Communication of benefits and harms associated with surgical and conservative care can optimize shared decision making. Further research should be conducted to evaluate for decisional regret and the impact of complications to inform treatment conversations.

**Supplementary Information:**

The online version contains supplementary material available at 10.1186/s41687-024-00810-z.

## Background

Cervical spine degeneration is a common condition with a prevalence approximated by patient’s age (i.e., 70% of 70 year-old patients) [1], that can lead to progressive neurologic dysfunction and disability. It is estimated that over 300,000 individuals in North America suffer from degenerative cervical myelopathy (DCM) which occurs when spondylotic changes compress the spinal cord [2, 3]. Symptoms may include fine motor dysfunction of the hands, upper extremity sensory changes, gait dysfunction and/or bladder/bowel incontinence.

The evidence informing the effectiveness of surgery vs. non-operative care for DCM is limited. A widely cited guideline from the AOSpine North America and the Cervical Spine Research Society (AO/CSRS) made strong recommendations for surgery in patients with moderate-to-severe DCM [4]. However, the strength of recommendations may be inconsistent with the available evidence which is primarily based on non-comparative studies. Weaker recommendations mandate shared decision-making (SDM) [5].

SDM is a process in which clinicians and patients jointly deliberate possible options for treatment and related risks so that the patient is well informed and makes a decision aligned with their preferences [6, 7]. Although considered a key element of high-quality care, SDM has been shown to be used to a limited extent in a variety of surgical disciplines [8]. The few studies in spine surgery demonstrate SDM to be inconsistently used [9–11]. Patient decision aids can be used to support SDM efforts, unfortunately, available educational resources for DCM are largely targeted at providers with little resources available for patients [39]. A potential barrier to the implementation of SDM in DCM is a lack of data on DCM patients’ values and preferences.

To address this knowledge gap, we conducted a study to evaluate various factors that may drive DCM patients’ treatment decisions. We applied a market research technique called a Discrete Choice Experiment (DCE) to formally quantify the relative importance of these factors [12, 13]. To determine whether the experience of cervical spine surgery impacts patient values and preferences, we conducted the DCE in two cohorts of cervical stenosis patients: those who had undergone previous surgery for DCM and those who had not.

## Methods

We adopted DCE methodology to determine the importance of (i) upper extremity neurologic function, (ii) lower extremity neurologic function, (iii) risk of future cervical spine surgery, (iv) dysphagia, and (v) C5 palsy. This research has been approved by the IRB of the authors’ affiliated institution.

DCEs, commonly used in product development for optimizing characteristics and pricing, are now increasingly applied in healthcare. For instance, they have helped explore the priorities in lumbar disc treatment from the perspectives of both surgeons and patients [14, 15]. In healthcare DCEs, participants are presented with pairs of hypothetical health states (choice sets) and asked to select their preferred state from each pair. Responses are analyzed using a variant of logistic regression to derive “importance scores” for the varying attributes in these scenarios.

**Fig. 1:**
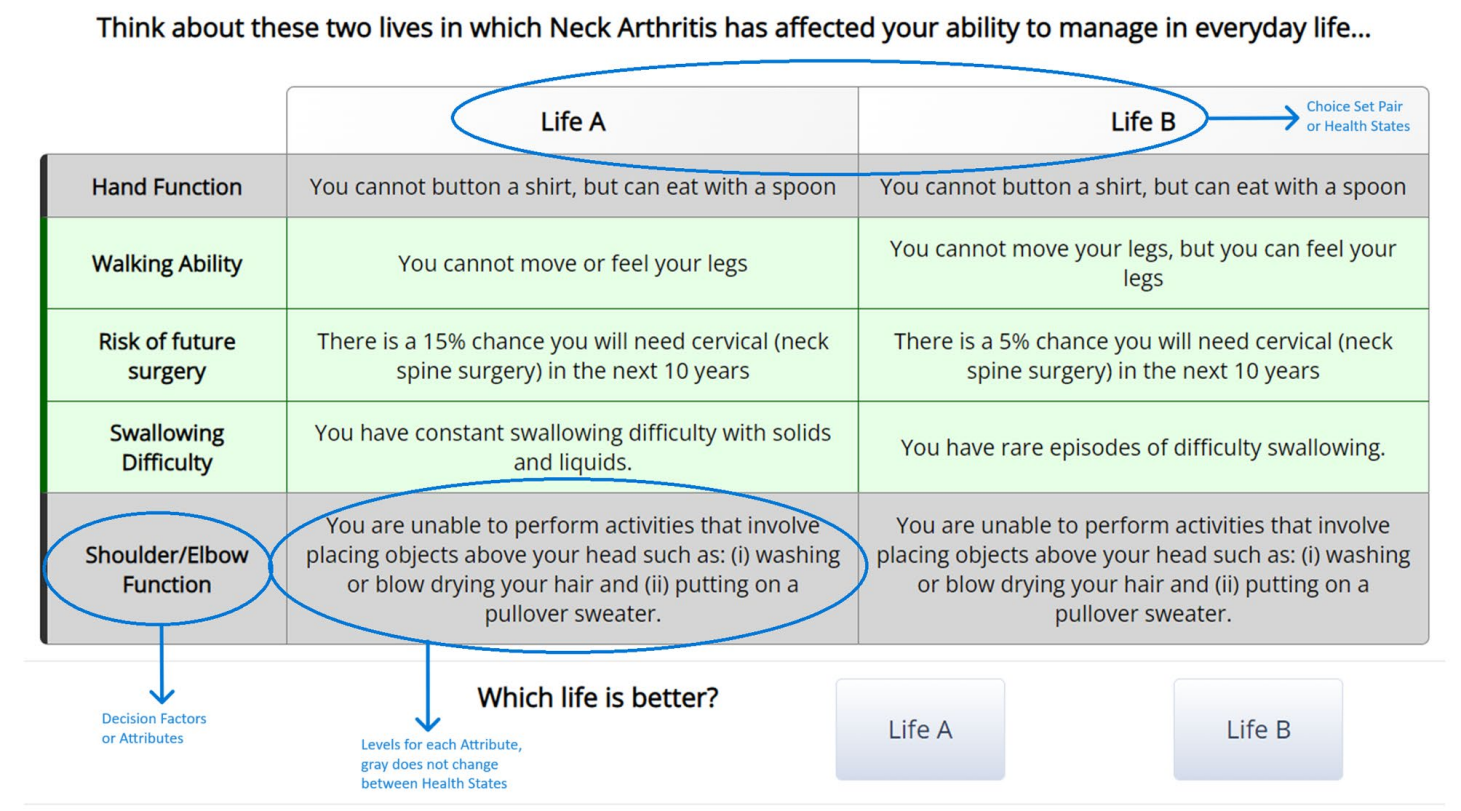
Choice set presented to patients

This approach allows for quantifying the relative importance of each factor in decision-making by using a collection of choice sets with attributes of varying severity. In our study, these sets were displayed in a table format, highlighting different attributes (Fig. 1) [16]. Coefficients were optimized for maximum ‘D-efficiency’ [17], a metric indicating the precision of coefficient estimates relative to the sample size and attribute number.

### Subjects

Our institution’s spine surgeons invited their patients being evaluated or followed up for Degenerative Cervical Myelopathy (DCM) or cervical spinal stenosis to participate in this study between March 2022 and September 2022. Additional inclusion criteria were: (1) age > 18 years old, and (2) the ability to read English. No patients were excluded based on the severity of DCM or whether they had undergone cervical spine surgery. To assess how previous surgery experiences influence patient preferences, we divided participants into two cohorts: those who had and had not undergone surgery for DCM. Non-operative patients were either awaiting surgery or being monitored clinically for mild myelopathy/cervical stenosis.

### Discrete choice experiment structure and task

Participants in the DCE were presented with pairs of health states (choice sets) as shown in Fig. 1 (annotations in blue text). Each health state comprised five attributes: (i) upper extremity neurologic function, (ii) lower extremity neurologic function, (iii) risk of cervical spine surgery, (iv) dysphagia, and (v) C5 palsy. We refer to these attributes as “decision factors.” We included neurologic function because this is the main indication for surgery in cervical myelopathy. We incorporated upper extremity and lower neurologic extremity function into the DCE because these are the most important sub-scores of the mJOA, reflected by the greatest number of points allocated [18]. We considered dysphagia and C5 palsy because these are the most common adverse events reported in RCTs on cervical myelopathy [19, 20]. We included risk of future surgery as a decision factor because approximately 20% of surgical patients undergo another cervical operation within 10 years [21]. Revision surgeries carry a greater risk of complications [22], and are less durable than primary procedures [23].

Upper and lower extremity neurologic function was described using items from the patient-derived version of the modified Japanese Orthopaedic Association (mJOA) score [24]. The mJOA is used to categorize patients with mild (15–17), moderate (12–14) or severe (0–11) neurologic deficits. Descriptions for dysphagia were adapted from the Bazaz Dysphagia Scoring System [25]. C5 function was described as per the shoulder function items from the Disabilities of the Arm, Shoulder and Hand (DASH) questionnaire [26]. We considered plausible 10-year revision rates with a 30% range centered on 20% [21]. Phrasing was in the second person and structured as declarative sentences [18, 27–29]. Decision factors and levels are shown in Table 1.

**Table 1:** Health state attributes and levels

Attribute Level	Arms	Legs	Risk of Revision Surgery	Dysphagia	C5 Palsy
0	You cannot move your hands	You cannot move or feel your legs	There is a 35% chance you will need cervical (neck spine surgery) in the next 10 years	You have constant swallowing difficulty with solids and liquids.	You are unable to perform activities that involve placing objects above your head such as: (i) washing or blow drying your hair and (ii) putting on a pullover sweater.
1	You cannot eat with a spoon, but can move your hands	You cannot move your legs, but you can feel your legs	There is a 25% chance you will need cervical (neck spine surgery) in the next 10 years	You have occasional swallowing difficulty with solid foods.	You will have severe difficulty with activities that involve placing objects above your head such as: (i) washing or blow drying your hair and (ii) putting on a pullover sweater.
2	You cannot button a shirt, but can eat with a spoon	You cannot walk, but you can move and feel your legs	There is a 15% chance you will need cervical (neck spine surgery) in the next 10 years	You have rare episodes of difficulty swallowing.	You have moderate difficulty with activities that involve placing objects above your head such as: (i) washing or blow drying your hair and (ii) putting on a pullover sweater.
3	It is very difficult for you to button a shirt	You can walk on flat ground when you use a cane or walker	There is a 5% chance you will need cervical (neck spine surgery) in the next 10 years	You have no episodes of difficulty swallowing	You have mild difficulty with activities that involve placing objects above your head such as: (i) washing or blow drying your hair and (ii) putting on a pullover sweater.
4	It is slightly difficult for you to button a shirt, and you frequently drop things	You can walk on flat ground without a cane or walker, but you need a handrail when using stairs	N/A	N/A	You have no difficulty with activities that involve placing objects above your head such as: (i) washing or blow drying your hair and (ii) putting on a pullover sweater.
5	Your hands work normally	You often lose your balance, but you do not need a cane or walker, and you do not need to use a handrail on stairs	N/A	N/A	N/A
6	N/A	You sometimes lose your balance, but you do not need a cane or walker, and you do not need to use a handrail on stairs	N/A	N/A	N/A
7	N/A	You walk normally	N/A	N/A	N/A

Although attribute levels may change over time (in particular: dysphagia and C5 palsy), it was not possible to incorporate this due to the interpretability of health state descriptions and increasing sample size requirements. Furthermore, other attributes are relevant to DCM treatment decision making (for example: pain, loss of motion, and acute spinal cord injury). Unfortunately, increasing number of attributes (i) requires an exponentially increasing sample size to produce a D-efficient choice set selection, and (ii) increases the cognitive burden of the exercise for participants [30, 31]. Therefore, due to methodological constraints, we selected the most important functional attributes in DCM [18] and the most common complications [19, 20] as attributes in the DCE.

### Survey procedures

A research assistant contacted potential participants by phone, explained the study, and obtained both verbal and electronic consent. After consenting, patients provided demographic information and their cervical spine treatment history. No incentives were offered for survey participation, and the survey was conducted by individuals (authors MM or MS) not directly involved in the participants’ care. Participants had the option to withdraw from the study at any point.

Upon logging into the survey, participants received information about cervical myelopathy and both surgical and non-surgical treatment options. They were then asked to answer two training questions, including one with an obviously superior health state. If a participant answered the control question incorrectly, they received feedback and could retry until they answered correctly. Following this, they completed ten DCE questions. At the survey’s conclusion, participants were asked to provide a rating on a five-point Likert scale (ranging from strongly disagree to strongly agree) for the statement: “This survey was difficult.”

Additionally, all patient charts were reviewed to identify post-operative complications.

### Choice set selection

As there exist over 700 000 unique choice sets, it was necessary to select a manageable subset for this study. We chose a D-efficient collection of 40 non-dominated choice sets, which we divided into blocks of 4 using the modified Federov algorithm with Ngene software (Supplementary Table S1) [30, 31]. The survey incorporated three levels of randomization: firstly, participants were randomly assigned to one of four blocks; secondly, the order of choice sets within each block was randomized; and thirdly, the order of health states was randomized for each participant.

### Statistical analysis

A utility function was estimated from DCE responses using a mixed multinomial-logit regression model using the “mlogit” library in the statistical programming language R [32, 33]. Each parameter was treated as a random effect to account for participant heterogeneity in the repeated DCE tasks. The random effects were modeled with 1000 draws from a normal distribution. In the regression model, DCE items were coded as ordinal variables to account for the severity of dysfunction. Model performance was evaluated using McFadden’s ρ^2^ [34]. Values between 0.2 and 0.4 indicate a very good model fit and are analogous to an R^2^ value between 0.7 and 0.9 for linear regression.

The mixed multinomial logit regression model was coded with an interaction effect for patients who had undergone surgery. The linear function derived from the regression coefficients yielded the absolute log-utility of a health state (utility is a measure of the desirability of a health state) [35]. The ratio of the absolute utility for health states in a choice set is the probability of selecting a given health state in a DCE and thus the ratios of regression coefficients quantify the relative importance of a decision factor.

S-efficiency is a measure of the minimum sample size to estimate statistically significant regression for DCE parameters at the 95% level [17]. Based on S-efficiency, the minimum sample size for the DCE design using the categorical model (more conservative assumption) was 100 participants (Table S1). We approached eligible patients consecutively until the desired sample size was reached.

## Results

101 individuals were invited to participate, and 100 provided consent to participate. Approximately half (*n* = 54) had previously undergone surgery for DCM, 55% were male, the mean age was 60.5 years, and the mean mJOA score was 13.8/18 (Table 2). Patients who underwent surgery tended to be male, older, and had a lower mJOA score. The mean mJOA score for the non-surgical group corresponded to mild severity (15.0/18), while for the surgical group the mean mJOA score corresponded to moderate severity (12.7/18). Most participants (65%) disagreed with the statement that “this survey was difficult.” 19% of (10 of 54) surgical patients experienced a post-operative adverse event (Table 2).

**Table 2:** Participant demographics and characteristics

			Pooled (N = 100)	Non-Surgical (N = 46)	Surgical (N = 54)
**Age**,** years**^**1**^			60.5 (21–84)	56.1 (21–84)	64.3 (33–82)
**Sex**					
	Female		44 (44.5%)	21 (45.7%)	23 (42.6%)
	Male		56 (55.4%)	25 (54.3%)	31 (57.4%)
**mJOA Score** ^**2**^					
	Upper Extremity		4.13 (1.2)	4.57 (0.7)	3.74 (1.4)
	Lower Extremity		4.85 (1.7)	5.62 (1.4)	4.18 (1.5)
	Sensory		2.22 (0.6)	2.15 (0.6)	2.28 (0.7)
	Sphincter		2.55 (0.7)	2.66 (0.5)	2.46 (0.8)
	Total		13.75 (3.0)	15.0 (2.1)	12.67 (3.3)
**Surgical Treatment**					
	Anterior cervical discectomy and fusion or cervical disc arthroplasty				21 (39%)
	Posterior laminectomy and fusion				27 (50%)
	Anterior corpectomy and fusion				5 (9%)
	Laminoplasty				1 (2%)
**Surgical Complications**					
	Dysphagia				
		Bazaz 1			2 (4%)
		Bazaz 2			0
		Bazaz 3			1 (2%)
	C5 Palsy				2 (4%)
	Deep wound infection				2 (4%)
	Hardware Failure				1 (2%)
	Hematoma				1 (2%)
	Worsening Myelopathy				1 (2%)
	Second Surgery				6 (11%)

^1^ mean (range)

^2^mean (standard deviation)

In the pooled analysis of non-surgical and surgical patients (Table 3), the rank order of decision factor coefficients for the non-surgical group was lower extremity neurologic function > upper extremity neurologic function > dysphagia > revision surgery > C5 palsy, whereas for the surgical group, revision surgery was the least important decision factor. The difference between lower and upper extremity neurologic function coefficients was statistically significant (*p* < 0.02, suggesting a difference in factor importance). However, there was no statistically significant difference between upper extremity neurologic function and dysphagia coefficients (*p* = 0.07) indicating that neurologic function does not trump potential complications as a decision factor. There was a statistically significant difference between dysphagia and revision surgery (*p* = 0.05), and dysphagia and C5 palsy (*p* = 0.05) indicating that dysphagia was the most influential complication-related decision factor. Differences in rank order between the surgical and non-surgical groups were not statistically significant (interaction coefficients for complications were not statistically significant). McFadden’s ρ^2^ indicated a very good fit for the model, at 0.35.

**Table 3:** Mixed multinomial-logit regression results

Health State Attribute		Decision Factor Coefficient Estimate	SE (Standard error)	$\:\varvec{z}$-value	*p*-value
*Pooled*					
	Loss of Lower Extremity Neurologic Function	-3.91	0.38	-10.42	< 0.0001
	Loss of Upper Extremity Neurologic Function	-2.53	0.27	-9.51	< 0.0001
	Experiencing Dysphagia	-1.69	0.28	-6.02	< 0.0001
	Needing Revision Surgery	-0.71	0.26	-2.70	0.007
	Experiencing a C5 Palsy	-0.72	0.22	-3.35	0.0008
*Non-Surgical*					
	Loss of Lower Extremity Neurologic Function	-4.10	0.41	-8.94	< 0.0001
	Loss of Upper Extremity Neurologic Function	-2.67	0.30	-10.05	< 0.0001
	Experiencing Dysphagia	-1.70	0.34	-5.00	< 0.0001
	Needing Revision Surgery	-0.60	0.29	-2.05	0.04
	Experiencing a C5 Palsy	-0.56	2.44	-2.29	0.02
*Surgical*					
	Loss of Lower Extremity Neurologic Function	-3.72	0.40	-9.36	< 0.0001
	Loss of Upper Extremity Neurologic Function	-1.83	0.32	-5.75	< 0.0001
	Experiencing Dysphagia	-1.76	0.36	-4.90	< 0.0001
	Needing Revision Surgery	-1.21	0.29	-4.14	< 0.0001
	Experiencing a C5 Palsy	-0.90	0.34	-2.66	0.03

From Table 3, we can see that the dispersion between the most important and least important decision factor in the non-surgical group (-4.10 to -0.56) was greater than that for the surgical group (-3.72 to -0.90). This indicates that overall, non-surgical patients were more strongly influenced by changes in neurology than surgical patients. While surgical patients were more strongly influenced by complications than non-surgical patients. However, these differences were only statistically significant for upper extremity neurologic function (*p* < 0.02), and approached statistical significance for C5 palsy (*p* < 0.06).

## Discussion

This study provides a new perspective—that of the patient—on treatment decisions for DCM. Our aim was to assess various factors influencing DCM patients’ treatment choices. Employing Discrete Choice Experiment (DCE) methodology, we quantified the significance of (i) upper extremity neurologic function, (ii) lower extremity neurologic function, (iii) risk of revision surgery, (iv) dysphagia, and (v) C5 palsy in these decisions. Key findings include:

i. Both patient groups deemed lower extremity neurologic function as the most crucial factor in treatment decisions.
ii. No significant statistical difference was found between the factors of dysphagia (a complication) and upper extremity neurologic function.
iii. Patients who underwent surgery prioritized neurologic function as less important and complications as more important compared to those who hadn’t undergone surgery.

Strengths of our study include use of a validated market research methodology to infer patient values from their expressed preference for health states [36]. Further, our regression model’s performance (McFadden’s ρ^2^ of 0.35) validates our modelling technique. Our surgical cohort was comparable to other published DCM cohorts in terms of ratio of anterior to posterior procedures and complications rate [37].

Shared Decision Making (SDM) is a collaborative process involving the clinician and patient, discussing treatment options, benefits, and harms to reach a decision that aligns with the patient’s values and preferences [38]. Previous research in spine surgery suggests deficient exploration of potential harms in SDM discussions [9, 10, 39]. Our findings underscore the importance of harms to patients, ranking them as crucial as certain neurological outcomes. Therefore, a values-based decision cannot be made without focused discussion of these aspects during the SDM discussion. Table 3 can help clinicians understand patients’ risk-benefit perceptions. For example, for patients who haven’t undergone surgery, lower extremity neurologic function is 1.5 times more important than dysphagia, while there is no statistically significant difference between upper extremity neurologic function and dysphagia. This implies that a patient seeking to improve lower extremity function might be more willing to risk postoperative dysphagia compared to a patient seeking improvement in upper extremity function.

This study confirms the importance of neurologic function which is well accepted in current guidelines as a critical factor for surgical decision making, however, it also highlights the importance of other factors to patients; for example, showing that dysphagia as a decision factor is not statistically significantly different than neurologic function. This study may not be exhaustive of all important patient factors, such as pain, and this may demonstrate an additional gap between patients and clinicians in decision making. Another limitation of this study is the included patients’ mean age. Although it is reflective of the demographic that most experiences cervical myelopathy, our findings may not apply to younger patients. Additionally, advancing age is associated with increased gait dysfunction [40], which may partly explain our patients’ prioritization of lower extremity neurologic function, although there was no statistically significant difference in factor rank order between groups. The stark difference in the importance of revision surgery between the surgical and non-surgical group may reflect a fundamental inability of the patient to grasp the gravity of spinal surgery until they experience it or may represent a barrier in communication between surgeons and patients regarding the experience of surgery.

As a small sample from a single institution, our results are not prescriptive to individual patients, but rather are a tool to enrich SDM discussions and underscore the need to devote more effort to exploring potential harms with patients [9, 10, 39]. A corollary is that patients neurologic benefits may not be paramount to patients, which may reflect a different value set than that for clinicians [4].

An important limitation of our study is that we only considered five decision factors. Sphincter function, sensory changes, axial and extremity pain have previously been shown to be important attributes in degenerative cervical pathology [18, 29]. Our health states also did not incorporate acute spinal cord injury, which is a feared complication of non-surgical treatment. We did not incorporate this because it is rare, with incidence rates for hospitalization between 13.9 per 1000 person-years [41]. This limitation is to allow for a D-efficient collection without exponentially increasing sample size, and to limit subject cognitive burden. Therefore, we selected the most important functional attributes in DCM [18]. and the most common complications [19, 20] as attributes in the DCE.

Further research should be undertaken to better understand how patient’s perceptions change after surgery. Our finding that post-operative patients weigh neurologic function as less important, and complications as more important than patients who have not undergone surgery suggest the potential for “decisional regret” [42] which if present would shape SDM discussions. We were not powered to study the relationship between complications and preferences, but this aspect should be explored in future work.

## Conclusions

This study is the first to inform us on patient’s perspective on treatment factors for DCM. Patient preferences for management of DCM are influenced by several considerations, and clear communication of benefits and harms associated with surgical and conservative care will help optimize patient-centered care. Further research should be conducted to evaluate for decisional regret and the impact of complications so that this information can be shared with patients during treatment conversations.

## Electronic supplementary material

Below is the link to the electronic supplementary material.

**Supplementary Material 1: Table S1** Discrete Choice Experiment Design

## Data Availability

The datasets generated and/or analysed during the current study are not publicly available, but are available from the corresponding author on reasonable request.

## Abbreviations

DCM: Degenerative cervical myelopathy
mJOA: Modified Japanese Orthopedic Association
CSRS: Cervical Spine Research Society
SDM: Shared decision-making
DASH: Disabilities of the Arm, Shoulder and Hand
