# Supplementary material for: Treatment decision-making factors among patients with cervical myelopathy: a discrete-choice experiment
Source: J Patient Rep Outcomes. 2024 Nov 11;8:129. doi: 10.1186/s41687-024-00810-z (PMC11554993; doi:10.1186/s41687-024-00810-z)
Supplement: Supplementary file 1 — Supplementary Material 1: Table S1 Discrete Choice Experiment Design [file 41687_2024_810_MOESM1_ESM.docx]

**Supplemental Table 1. Discrete Choice Experiment Design**

|  |  | **Alternative 1** | | | | |  | **Alternative 2** | | | | |
| --- | --- | --- | --- | --- | --- | --- | --- | --- | --- | --- | --- | --- |
| **Choice Set** | **Block** | **Arms** | **Legs** | **Risk of Revision** | **Dysphagia** | **C5 Palsy** |  | **Arms** | **Legs** | **Risk of Revision** | **Dysphagia** | **C5 Palsy** |
| 1 | 1 | 0 | 5 | 0 | 3 | 2 |  | 4 | 3 | 0 | 3 | 0 |
| 2 | 1 | 4 | 5 | 1 | 2 | 0 |  | 2 | 2 | 1 | 2 | 3 |
| 3 | 3 | 0 | 2 | 1 | 0 | 4 |  | 1 | 2 | 0 | 0 | 0 |
| 4 | 2 | 4 | 7 | 2 | 3 | 4 |  | 4 | 0 | 3 | 3 | 3 |
| 5 | 1 | 1 | 5 | 3 | 1 | 2 |  | 5 | 3 | 3 | 2 | 2 |
| 6 | 1 | 5 | 0 | 1 | 2 | 3 |  | 2 | 2 | 1 | 2 | 0 |
| 7 | 3 | 4 | 2 | 0 | 0 | 2 |  | 3 | 4 | 3 | 0 | 2 |
| 8 | 4 | 2 | 0 | 2 | 0 | 0 |  | 2 | 1 | 3 | 2 | 0 |
| 9 | 3 | 2 | 2 | 1 | 3 | 2 |  | 1 | 2 | 1 | 2 | 4 |
| 10 | 1 | 0 | 4 | 2 | 1 | 1 |  | 1 | 7 | 2 | 1 | 3 |
| 11 | 4 | 0 | 6 | 0 | 1 | 1 |  | 0 | 1 | 2 | 1 | 0 |
| 12 | 3 | 5 | 1 | 2 | 3 | 4 |  | 5 | 7 | 2 | 0 | 0 |
| 13 | 2 | 0 | 2 | 2 | 3 | 3 |  | 2 | 6 | 2 | 3 | 4 |
| 14 | 4 | 0 | 3 | 2 | 3 | 4 |  | 5 | 1 | 2 | 3 | 1 |
| 15 | 2 | 2 | 5 | 0 | 3 | 1 |  | 2 | 4 | 1 | 3 | 0 |
| 16 | 1 | 2 | 1 | 1 | 0 | 0 |  | 5 | 2 | 2 | 0 | 0 |
| 17 | 4 | 1 | 6 | 0 | 2 | 2 |  | 4 | 6 | 0 | 3 | 3 |
| 18 | 4 | 0 | 5 | 2 | 3 | 0 |  | 2 | 7 | 2 | 1 | 0 |
| 19 | 4 | 2 | 6 | 2 | 0 | 0 |  | 1 | 4 | 2 | 0 | 2 |
| 20 | 4 | 1 | 5 | 0 | 3 | 3 |  | 5 | 5 | 0 | 1 | 2 |
| 21 | 1 | 1 | 3 | 3 | 1 | 2 |  | 1 | 4 | 3 | 2 | 0 |
| 22 | 1 | 3 | 0 | 0 | 1 | 2 |  | 5 | 4 | 0 | 1 | 3 |
| 23 | 2 | 1 | 2 | 1 | 0 | 3 |  | 0 | 7 | 2 | 0 | 3 |
| 24 | 3 | 5 | 6 | 3 | 3 | 2 |  | 2 | 0 | 0 | 3 | 2 |
| 25 | 2 | 0 | 6 | 0 | 2 | 2 |  | 5 | 5 | 1 | 2 | 2 |
| 26 | 2 | 2 | 3 | 3 | 1 | 1 |  | 2 | 3 | 0 | 0 | 2 |
| 27 | 2 | 2 | 6 | 2 | 3 | 2 |  | 3 | 1 | 2 | 1 | 2 |
| 28 | 4 | 3 | 3 | 1 | 1 | 1 |  | 4 | 0 | 1 | 1 | 2 |
| 29 | 3 | 1 | 0 | 0 | 1 | 3 |  | 3 | 0 | 3 | 0 | 3 |
| 30 | 3 | 3 | 6 | 1 | 3 | 4 |  | 2 | 5 | 1 | 1 | 4 |
| 31 | 2 | 3 | 2 | 1 | 0 | 1 |  | 1 | 3 | 2 | 0 | 1 |
| 32 | 3 | 4 | 1 | 3 | 0 | 2 |  | 5 | 5 | 3 | 0 | 3 |
| 33 | 2 | 5 | 7 | 3 | 3 | 1 |  | 5 | 1 | 3 | 0 | 4 |
| 34 | 1 | 0 | 0 | 1 | 2 | 3 |  | 1 | 4 | 1 | 0 | 3 |
| 35 | 3 | 1 | 2 | 2 | 3 | 2 |  | 4 | 3 | 0 | 3 | 2 |
| 36 | 1 | 2 | 4 | 1 | 1 | 4 |  | 2 | 3 | 1 | 0 | 1 |
| 37 | 3 | 1 | 0 | 2 | 0 | 1 |  | 3 | 0 | 2 | 3 | 0 |
| 38 | 4 | 3 | 0 | 0 | 1 | 1 |  | 4 | 7 | 3 | 1 | 1 |
| 39 | 4 | 4 | 0 | 3 | 2 | 3 |  | 0 | 3 | 1 | 2 | 3 |
| 40 | 2 | 3 | 7 | 3 | 2 | 1 |  | 0 | 6 | 3 | 2 | 0 |
